# Polyorchidism: A Case Report on Diagnostic Approach and Surgical Considerations

**DOI:** 10.1155/criu/5686215

**Published:** 2026-04-30

**Authors:** Ali Alyami, Ebtesam Almajed, Ahmed AlShammari, Tariq Burki

**Affiliations:** ^1^ Department of Pediatric Urology, King Abdullah Specialized Children Hospital, Ministry of National Guard - Health Affairs, Riyadh, Saudi Arabia

**Keywords:** case report, polyorchidism, testicular atrophy, triorchidism

## Abstract

Polyorchidism is a rare developmental anomaly in which more than two testes are present, usually diagnosed incidentally through imaging. The most frequent presentation is triorchidism, with the supernumerary testis typically located within the scrotum or inguinal canal. Embryologically, the condition is attributed to abnormal partitioning of the genital ridge during early development, specifically before the eighth week of gestation. Ultrasonography remains the initial imaging modality of choice, with MRI providing further diagnostic clarity if needed. We report the case of a 4‐year‐old male with a history of right cryptorchidism and prior orchidopexy, in whom left‐sided polyorchidism was incidentally detected during ultrasound assessment of an atrophic right testis. Management is determined by associated anomalies and the risk of malignancy; conservative surveillance is generally recommended for morphologically normal, scrotal testes, whereas surgical intervention, including orchiectomy, is reserved for extrascrotal locations, atrophic gonads, or when malignancy is suspected.

## 1. Introduction

Polyorchidism refers to a rare developmental anomaly in which more than two testes are present, which may be located within the scrotum or ectopically. Polyorchidism, also referred to as a supernumerary testis, is an exceptionally rare congenital anomaly, with fewer than 200 cases documented in the literature [1]. In a systematic review by Balawender et al., conducted between 2000 and 2021, a total of 64 patients with polyorchidism were identified [2].

The most frequent variant, triorchidism, involves three testes [2]. The embryogenesis of polyorchidism is thought to result from abnormal division of the germinal ridge before the eighth week of gestation [3]. Two main hypotheses have been proposed: transverse or longitudinal duplication of the genital ridge and primordial gonad, both of which can lead to supernumerary testicular formation [1]. Clinically, most patients are asymptomatic, with the condition often discovered incidentally as a palpable scrotal mass. Ultrasound serves as the primary diagnostic modality, offering characteristic features that aid identification, whereas magnetic resonance imaging (MRI) may provide additional diagnostic confirmation [4]. Here, we report the case of a 4‐year‐old male in whom polyorchidism was incidentally diagnosed during a routine testicular ultrasound.

## 2. Case Description

A 4‐year‐old boy was referred to pediatric urology with suspected testicular duplication after clinical examination revealed an unusual palpable structure adjacent to the left testis during follow‐up evaluation. He had a known history of right undescended testis for which he underwent diagnostic laparoscopy followed by single‐stage laparoscopic‐assisted right orchidopexy for a peeping testis at the age of 16 months. Intraoperatively, the vas and vessels were seen entering the left deep inguinal ring, and a small patent processus vaginalis was noted but left untreated. At 33 months of age, he required a redo right inguinal orchidopexy due to testicular re‐ascent.

**FIGURE 1:**
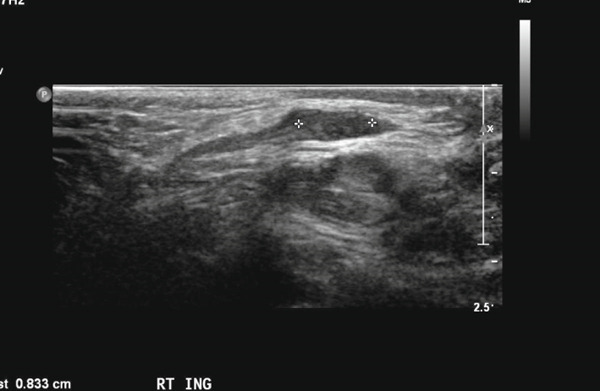
Ultrasound of the right inguinal canal demonstrates a small, hypoechoic testis measuring 0.83 cm with absent internal vascularity, consistent with testicular atrophy.

**FIGURE 2:**
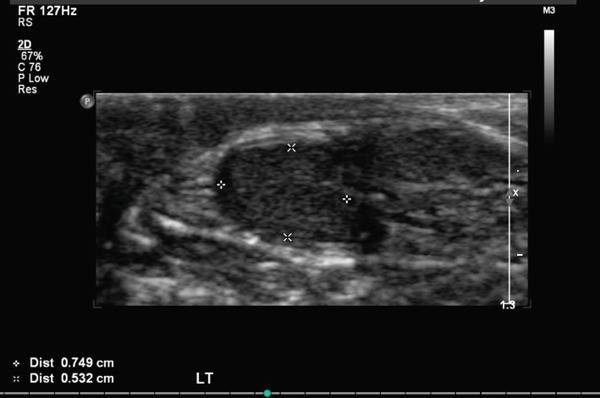
Ultrasound of the left hemiscrotum showing a normal testis.

**FIGURE 3:**
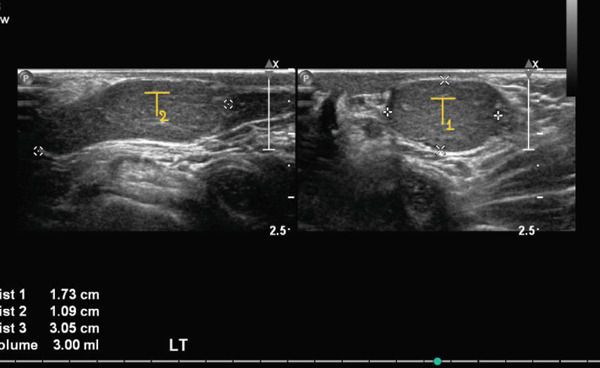
Ultrasound image of the left hemiscrotum demonstrating two testes. The smaller intrascrotal testis (T1) represents the normal left testis, whereas the larger superior testis (T2), located partially within the inguinal region, represents the supernumerary testis. Both structures demonstrate normal echogenicity and preserved vascularity.

Subsequent follow‐up revealed a small right testis and an enlarged left testis, prompting ultrasonography to exclude an underlying testicular mass. Ultrasound demonstrated a hypoechoic, small right testis located in the inguinal canal, measuring 0.4 × 0.3 × 0.8 cm, with absent internal vascularity, consistent with atrophy (Figure 1). On the left side, two testes were visualized: one intrascrotal and the other positioned higher, partially within the inguinal region, measuring 0.7 × 0.5 × 1.1 cm and 1.7 × 1.1 × 3.0 cm, respectively (Figure 2). Both left‐sided testes demonstrated normal echogenicity with preserved vascularity, findings compatible with polyorchidism (Figure 3; T1 = normal intrascrotal testis, T2 = supernumerary testis located partially in the inguinal region). On clinical examination, an atrophic testis was palpable on the right side. On the left side, a normal testis with a firm oval structure adherent to its lower pole was identified. A diagnosis of right testicular atrophy with left‐sided polyorchidism was made. Given the absence of symptoms, atrophic right testis, and reassuring imaging, the patient was managed conservatively with a plan for annual ultrasonographic surveillance.

## 3. Discussion

Polyorchidism is an exceptionally rare congenital anomaly, most often presenting as triorchidism. In a recent systematic review, approximately 76% of supernumerary testes were located in the scrotum and 24% were extrascrotal, with the majority of the latter found in the inguinal canal (87%) and fewer in the abdominal cavity (13%) [2]. The anatomical findings in the present case, namely left‐sided polyorchidism with one supernumerary testis located partially within the inguinal region, are therefore consistent with the most commonly described patterns in pediatric patients. The extra testis has a left‐sided predominance and is frequently found either superior or inferior to the normal testis [5].

Initially, the primordial testis develops from the primitive genital ridge, located medial to the mesonephric ducts. Under the influence of the SRY gene, this primordial testis develops rete testis and seminiferous tubules and connects to the epididymis and the ductus deferens. The various types of polyorchidism can be explained by the duplication of the genital ridge and the mesonephric ducts that happens horizontally or longitudinally. Based on embryological development, Leung proposed a four‐type classification of polyorchidism [6]. Type I refers to a supernumerary testis without an epididymis or vas deferens, believed to result from division of the genital ridge without contact with the rete testis. In Type II, the supernumerary testis has its own epididymis but shares a vas deferens, a consequence of the complete division of the genital ridge and mesonephros. In Type III, the supernumerary testis and the normal testis share both an epididymis and a vas deferens, as only the genital ridge divides while the mesonephros remains intact; this is the most common form [7]. Type IV represents complete duplication of the testis, epididymis, and vas deferens, arising from simultaneous duplication of both the genital ridge and mesonephros. In the present case, the supernumerary testis appeared attached to the lower pole of the normal testis and demonstrated preserved echogenicity and vascularity on ultrasonography. Based on the embryological classification proposed by Leung, this presentation is most consistent with Type III polyorchidism, in which the supernumerary testis shares the epididymis and vas deferens with the ipsilateral normal testis. According to the functional classification described by Bergholz and Wenke, this case likely corresponds to Type A polyorchidism, indicating the presence of a drainage pathway through the epididymis and vas deferens. These classifications suggest that the supernumerary testis may have reproductive potential and support a conservative management approach when imaging findings are reassuring.

As with other scrotal pathologies, the initial diagnostic modality of choice is ultrasonography, which has a reported sensitivity of 80%–95% [8]. It typically demonstrates a normal testis with preserved echotexture and vascularity. In contrast, the supernumerary testis may be located superior or inferior to the ipsilateral testis, either attached to or separate from it. When ultrasound findings are inconclusive, MRI provides additional diagnostic value. On MRI, the supernumerary testis usually appears as a round or oval structure resembling a normal testis, with characteristic homogeneous intermediate signal intensity on T1‐weighted images and high signal intensity on T2‐weighted images [9].

The differential diagnosis of polyorchidism includes several scrotal and inguinal pathologies that may present as a palpable scrotal mass. These can be broadly categorized into intratesticular and extratesticular lesions. Intratesticular differentials include testicular tumors such as germ cell tumors and epidermoid cysts [10]. Extrapesticular lesions include benign masses such as adenomatoid tumors, lipomas of the spermatic cord, epididymal cysts, and varicoceles. Additionally, non‐neoplastic conditions such as inguinal hernia or hydrocele may clinically mimic a supernumerary testis. Polyorchidism carries a small increased risk of testicular cancer compared to the general population [2, 10]. Bergholz et al. reported that among 140 documented cases of polyorchidism, approximately 6% were associated with testicular neoplasms [11]. A recent systematic review reported a malignancy rate closer to 4% [2]. Most tumors are germ cell tumors, for example, seminoma arising in the supernumerary testis [2]. Importantly, most malignant transformations have been reported in undescended or extrascrotal supernumerary testes, particularly those located in the inguinal canal or abdomen. In contrast, scrotal supernumerary testes with normal imaging characteristics appear to carry a substantially lower risk of malignancy.

In clinical practice, management decisions in polyorchidism are influenced by both the anatomical location of the supernumerary testis and its potential reproductive function. Supernumerary testes located within the scrotum or inguinoscrotal region with preserved vascularity and normal morphology are more likely to have reproductive potential. In contrast, abdominal or markedly dysplastic supernumerary testes are less likely to contribute to fertility and may carry a higher risk of malignancy. Management of polyorchidism in children is individualized, balancing cancer risk with fertility potential. Key factors include the location, testicle′s viability, attached structures, and the child′s age [10, 12]. Conservative observation is often favored in pediatric cases when the supernumerary testis is scrotal, appears normal on imaging, and there are no concerning features [10]. By contrast, surgical intervention is advised if there are complicating factors. Exploration is warranted for any extrascrotal supernumerary testis or if complications like testicular torsion, significant atrophy, or suspicion of malignancy are present [10]. In such scenarios, intervention is done to either prevent torsion or obtain tissue diagnosis, as appropriate.

When surgery is performed, testis‐sparing strategies are often possible and preferred if the testis is viable. Historically, many supernumerary testes were removed prophylactically due to cancer concerns, but this approach has evolved [2]. Recent series demonstrate a trend toward preserving normal testicular tissue. According to a systematic review, 46% of patients were observed and 54% underwent surgery, of which 59% had orchidopexy instead of orchidectomy, reflecting a shift toward testis preservation when imaging shows a normal‐appearing testis [2]. Other than that, orchiectomy and biopsy of the lesion can be done in the presence of existing conditions such as cryptosporidium, testicular torsion, or suspicion of malignancy [12].

In a thorough review of the recent literature over the last 5 years, polyorchidism reports increasingly rely on high‐resolution ultrasound and selective MRI to justify testis‐preserving surveillance when imaging is benign, while reserving surgery for ectopic/cryptorchid testes, torsion, atrophy, or suspected/confirmed malignancy. A total of 28 primary case reports/series describe 29 patients age range among reports 2–53 years, with laterality reported as left‐predominant (12 left, 8 right, 3 bilateral, 6 unspecified) and pediatric presentations commonly linked to cryptorchidism or inguinal hernia/hydrocele discovered intra‐operatively during orchidopexy/hernia repair [13]. Across the studies, torsion remains the clearest trigger for urgent exploration with absent Doppler flow prompted orchiectomy of nonviable supernumerary gonads or detorsion with fixation when salvageable [14]. Indications for elective excision included severely atrophic/hypoplastic or nonscrotal testes found at exploration, parental preference for histologic confirmation, or oncologic concern; histopathology ranged from normal spermatogenesis/benign seminiferous tissue to benign atrophy and rarely seminoma in an intra‐abdominal mass [13].

The reported distribution of supernumerary testes also has practical implications for management in pediatric patients. Scrotal supernumerary testes, which account for approximately three‐quarters of reported cases, are generally considered lower risk when imaging demonstrates normal morphology and preserved vascularity. In such situations, conservative management with periodic clinical examination and ultrasonographic surveillance is typically recommended to preserve potential fertility and avoid unnecessary surgery. In contrast, extrascrotal supernumerary testes particularly those located within the inguinal canal or abdomen are associated with a higher theoretical risk of malignancy and torsion. These cases may warrant surgical exploration, with orchiopexy performed if the testis appears viable or orchiectomy considered when the gonad is atrophic, dysplastic, or suspicious for malignancy.

An important clinical consideration in polyorchidism is determining the threshold for surgical exploration versus conservative surveillance, particularly in pediatric patients. Historically, many supernumerary testes were removed prophylactically due to concerns regarding malignancy. However, advances in imaging modalities, particularly high‐resolution ultrasonography with Doppler assessment, have improved diagnostic confidence and allowed physicians to adopt a more conservative approach in selected cases. In the present case, the supernumerary testis demonstrated normal echogenicity and preserved vascularity on ultrasound, with no suspicious features. Therefore, surveillance with periodic ultrasonography was considered appropriate.

In patients managed conservatively, follow‐up typically includes periodic clinical examination and ultrasonographic surveillance. Annual ultrasonography is commonly recommended during childhood and adolescence to monitor the size, echotexture, and vascularity of the supernumerary testis. Surveillance aims to detect any early changes that may raise suspicion for malignancy or other complications. Findings that may prompt further evaluation include progressive enlargement, focal nodular lesions, heterogeneous echotexture, or abnormal Doppler vascularity. In such situations, additional imaging with MRI or surgical exploration may be considered to further characterize the lesion and guide management.

Although polyorchidism has been reported in the literature, each additional report contributes to improving understanding of the clinical spectrum and management strategies of this rare condition. The present case highlights the incidental detection of contralateral polyorchidism during surveillance following orchidopexy for cryptorchidism with subsequent testicular atrophy. This uncommon clinical scenario illustrates the importance of careful postoperative imaging and supports the role of conservative management when imaging demonstrates normal morphology and vascularity.

## 4. Conclusion

Polyorchidism is a rare anomaly with low malignancy risk. Ultrasonography serves as the primary diagnostic modality. Management should be individualized, with conservative surveillance favored for normal scrotal testes, whereas surgical intervention is reserved for extrascrotal, atrophic, torsed, or suspicious cases to balance safety and fertility.

## Author Contributions

Ebtesam Almajed had full access to all of the data in this study and takes complete responsibility for the integrity of the data and the accuracy of the data analysis.

## Funding

No funding was received for this manuscript.

## Disclosure

All authors have read and approved the final version of the manuscript.

## Ethics Statement

Ethical approval was not required for this case report according to institutional policy.

## Consent

Written informed consent was obtained from the patient′s legal guardian for publication of this case report and any accompanying images.

## Conflicts of Interest

The authors declare no conflicts of interest.

## Data Availability

The data supporting the findings of this study are included within the article. Additional information may be available from the corresponding author upon reasonable request.
